# Vaccination of Patients with Chronic Kidney Disease and Cocooning Strategy in a Tertiary Hospital

**DOI:** 10.3390/vaccines14080655

**Published:** 2026-07-26

**Authors:** Maria Michailou, Maria Bitsori, Kostas Stylianou, Diamantis Kofteridis, Evangelos Blevrakis, Rozalia Dimitriou, Maria Zacharioudaki, Emmanouil Galanakis

**Affiliations:** 1 Department of Paediatrics, Heraklion University Hospital, 71500 Crete, Greece; maraki3108@hotmail.com (M.M.); bitmar2@gmail.com (M.B.); maria.zacharioudaki@med.uoc.gr (M.Z.); 2 Department of Nephrology, Heraklion University Hospital, 71500 Crete, Greece; kstylianu@uoc.gr; 3 School of Medicine, University of Crete, 71409 Heraklion, Greece; kofterid@med.uoc.gr (D.K.); e.blevrakis@uoc.gr (E.B.); r.dimitriou@uoc.gr (R.D.); 4 Department of Internal Medicine, Heraklion University Hospital, 71500 Crete, Greece; 5 Department of Pediatric Surgery, Heraklion University Hospital, 71500 Crete, Greece; 6 Department of Orthopedic Surgery, Heraklion University Hospital, 71500 Crete, Greece

**Keywords:** vaccination, immunization, vaccination coverage, cocooning, chronic kidney disease, CKD

## Abstract

Background: Despite the increased infection-related morbidity of patients with chronic kidney disease (CKD), their vaccination coverage is low. Cocooning strategy has not been adequately researched in this group. Our aim was to evaluate vaccination coverage of CKD pediatric and adult patients and their families. Methods: In this prospective, single-center study, we recorded the vaccination coverage of CKD pediatric and adult patients and their families who attend a tertiary University Hospital. Vaccination rates were calculated according to the national vaccination program. Vaccination of pediatric CKD patients was compared to a control group of healthy children. Results: The vaccination rate of 63 hemodialysis patients was low for influenza (61.9%), COVID-19 (79.4% primary, 3.2% booster doses), RSV (50%), herpes zoster (42.4%), hepatitis B (41.3%), *Streptococcus pneumoniae* (25.4%), tetanus–pertussis (3.2%), and HPV (0%). Their 30 underaged relatives had low vaccination rates for influenza (13.3%), DTaP/Tdap (80%), and Men B (50%), with adequate vaccination for other pathogens. The 53 children with CKD were poorly vaccinated for influenza (20.8%), COVID-19 (0%), *Streptococcus pneumoniae* (15.4%), Men B (30.2%), DTaP/Tdap (67.9%), MMR (78.8%) and VZV (86.5%), but their vaccination rates for influenza were significantly higher compared to controls. Their 108 adult relatives were inadequately vaccinated for influenza (27.8%), COVID-19 (1.9%), and Tdap (1.9%). Their 35 underaged relatives were poorly vaccinated for influenza (22.9%) and DTaP (80%). Conclusions: Vaccination coverage of CKD patients is suboptimal for vaccines particularly important for their condition and their family members are insufficiently informed about potential contribution to their protection by cocooning strategy.

## 1. Introduction

Chronic kidney disease (CKD) is a major contributor in morbidity and mortality worldwide with a steady increase in prevalence in the past 30 years [1]. However, a large portion of patients are still underdiagnosed and under-documented, and our strategies of limiting the progression of the disease are inadequate [2,3,4]. These patients experience an increased rate of infection-related complications, with infections being the second most common cause of mortality after cardiovascular events [5,6,7,8]. Therefore, strategies for prevention of infections are vital for patients with CKD.

Immunization is an invaluable strategy for infection prevention with a proven efficacy in several vulnerable groups. The particular difficulties of patients with CKD in terms of management lie in their certain level of immunosuppression, especially in the later stages, which limits their response to vaccination, and their level of vaccine hesitancy associated with fear of rejection in the case of a kidney transplant or relapse in the case of nephrotic syndrome [9,10,11]. This leads to a decreased rate of vaccination, despite the patients’ susceptibility or the clearly stated need in guidelines [12].

Cocooning strategy is defined as the vaccination of vulnerable individuals’ close contacts, with the intent of protecting them from exposure to preventable infectious diseases [13]. The advantage of this approach is that it enhances vaccination rates through altruism and love, rather than strict implementations or other mandatory vaccination policies [14]. The utility of the strategy is highlighted in the case of air-borne highly transmissible pathogens such as influenza and COVID-19 and in pathogens that are prevented with live vaccines, which are contra-indicated in specific patient groups [12]. CKD patients experience increased morbidity and infectious complications such as increased mortality and hospitalization rates from these pathogens [5,15,16,17,18] and therefore could benefit from the implementation of the strategy. To our knowledge, there are no published surveys on implementation of cocooning strategy in CKD patient group.

The exploration of trends of vaccination within CKD patients and their environment is significantly important considering the historical, environmental and epidemiological changes that were noted in the last decade [19]. The introduction of new vaccination products, the emergence of a pandemic during 2020, the rise in hesitancy, or the increase in unvaccinated refugee populations could all potentially affect the vaccination coverage of susceptible groups such as CKD patients [20,21].

Our study was conducted in the island of Crete, a region of Greece where high rates of vaccination have been reported for the general pediatric population and intermediate for adults in previous scarce vaccine coverage studies, and similar observations are true for vulnerable groups [22,23,24,25,26]. Studies regarding cocooning strategy implementation revealed suboptimal vaccination of close contacts in our center [24,25]. However, a European study places Greece in the eighth place in vaccine skepticism amongst 67 countries [27]. The economic recession has not altered the policy of free vaccination and healthcare; therefore access to a healthcare unit is the major determinant for successful vaccination [28]. The refugee influx of the past decade, along with the pre-existing minority groups, increases the unvaccinated group due to limited access to healthcare or cultural issues [29,30]. All of the above could act as determinants of vaccination.

There is no official registry of children with CKD in our area, except for end-stage patients; thus the true prevalence of the condition is largely unknown. The estimated national prevalence of end-stage CKD (stage 5) in children is 20 per million of age-related population (pmarp) according to the European Society of Pediatric Nephrology (ESPN) registry [31]. Adult hemodialysis patient numbers in Crete were roughly estimated at around 220 patients in a previous study of 2017 [32]. Infection-related complications might lead to disease progression in this limited number of patients, which accounts for a disproportionate burden for the healthcare system [33]. Vaccination has proved to be a cost-effective measure of disease prevention [34,35], and the implementation of cocooning strategy could enhance that effect.

The aim of this study is to document the vaccination rates of specific subgroups of patients with CKD as well as the vaccination coverage of their close contacts in a single center. Furthermore, we investigate the potential effect of the COVID-19 pandemic on vaccination rates by comparing the data of these patients to pre-pandemic recorded vaccination coverage data. Through this study, we intend to provide insight into an important component in this patient group’s management, such as vaccination, which has not been adequately studied and is often overlooked by clinicians.

## 2. Materials and Methods

### 2.1. Study Design

**Figure 1:**
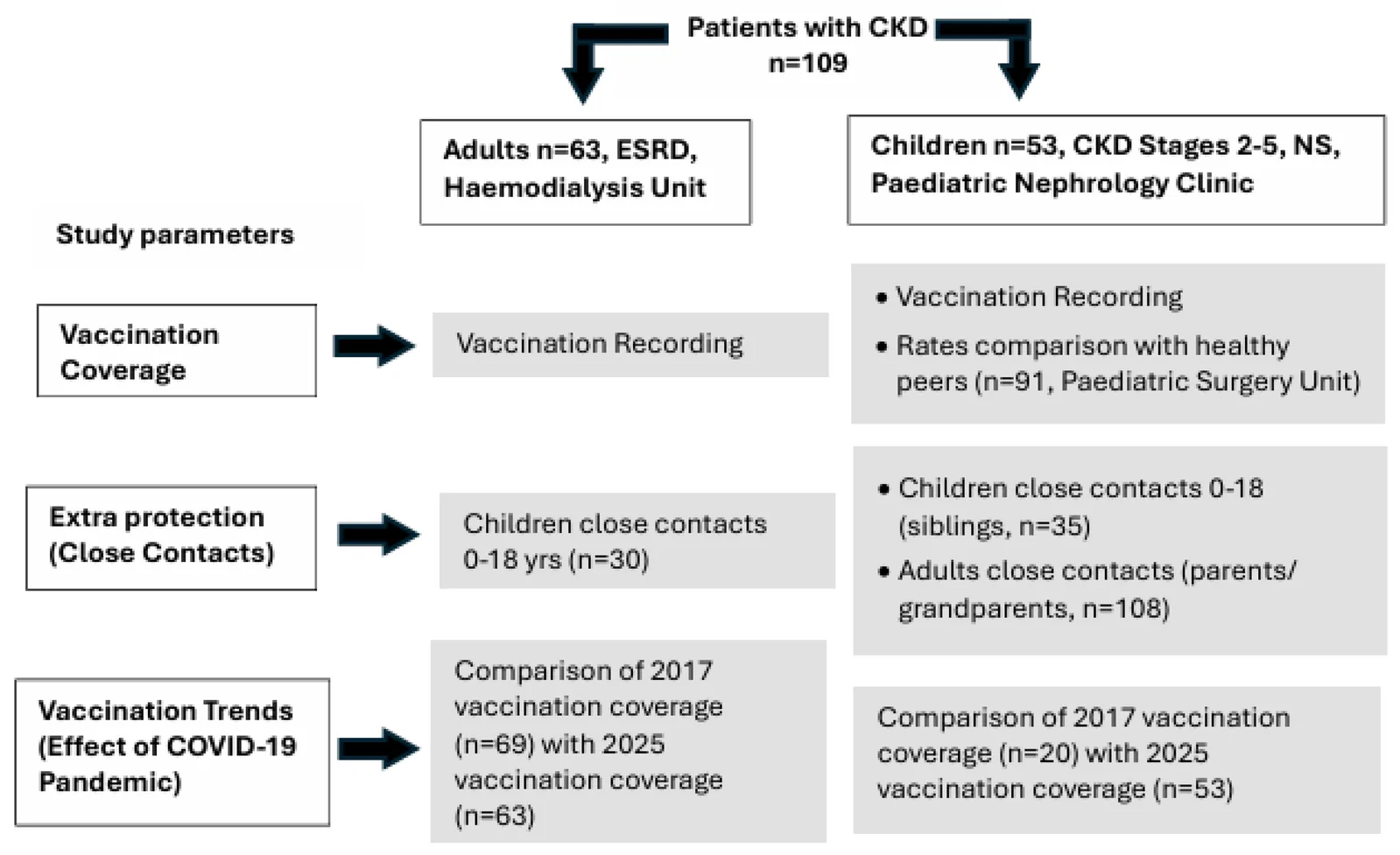
Flowchart of study population and study parameters.

This is a single-center cross-sectional study, which also includes a case–control comparison, that was conducted during July 2025–January 2026 in a tertiary University Hospital. The study investigated the vaccination rates of CKD patients and their close contacts to evaluate whether the cocooning strategy has a role in the further protection of this population. The adequacy of vaccination of children with CKD was further evaluated by comparison with the vaccination status of similarly aged, healthy children. To determine whether the COVID-19 pandemic affected vaccination trends in CKD patients and their relatives in our area of practice, the data from the present study were compared with the findings of a similar study in 2017 in the same area (Figure 1).

### 2.2. Study Population

The study included two groups of CKD patients: a group of adult patients undergoing dialysis at the University Hospital dialysis unit and pediatric patients with CKD who attend the Pediatric Nephrology Outpatient Clinic. The implementation of the cocooning strategy was evaluated by recording the vaccination rates of close contacts, who were children aged 0–18 years for adult dialysis patients and both adults and children aged 0–18 years for children with CKD. The control group consisted of children attending the Pediatric Surgery Clinic.

Enrollment was prospective; participants were approached upon hospital visit or admission, and interviews were conducted either in person or by telephone. Vaccinations were documented based on the participant’s claim and cross-checked with pediatric booklets, the pediatric vaccination registry, the electronic prescription system, and the clinic or hemodialysis unit archive. We included every vaccination until the day of the interview and did not count intention to get vaccinated or future vaccinations within the time of the study.

### 2.3. Eligibility Criteria

The adult CKD group included patients from the hemodialysis unit under regular hemodialysis. The pediatric patient group included all children (0–18 years) with CKD stages 2–5 and children with nephrotic syndrome or other chronic renal conditions who are followed by the Pediatric Nephrology Outpatient Clinic. Close contact groups included children with frequent contact with adult dialysis patients and parents/grandparents and siblings of children with CKD.

Every child with CKD was matched with at least 2 children of similar age and the same gender, without any renal or other chronic health conditions, who were recruited from the Pediatric Surgery Department and served as a control group for the assessment of the vaccination status of children with CKD (Figure 1). Thus, eligibility criteria included sex and age matching to an enrolled child with CKD and admission to the Pediatric Surgery Clinic at the time of the study, and criteria for exclusion included known renal or other chronic health condition. No geographical or socioeconomic criteria were enforced upon selection.

### 2.4. Variables

Epidemiological and vaccination data were curated from interviews with the patients and their relatives/caretakers. The participants consented to be included in the study after they were informed of its purpose, the use of their personal data, and the prospect of future publication of the results of the research. The interview-obtained data were cross-checked with the archives of the clinics and with the electronic prescription system, as well as the vaccination booklets for pediatric patients. The adequacy of vaccination was assessed according to the Greek Vaccination Program for Adult Patients of 2025 and the Greek Vaccination Program for Children and Adolescents of 2025 (Table 1).

Vaccination status was assessed either by dividing participants into vaccinated and unvaccinated groups or, in the case of children with CKD, by dividing them into adequately vaccinated or unvaccinated/incompletely vaccinated groups based on the number of doses (according to their age). The participants who were considered adequately vaccinated had received every recommended dose of a vaccine. In the case of vaccines that are recommended only for a subgroup of our patients based on their age, such as human papillomavirus (HPV) and *Streptococcus pneumonia* (PCV), the percentage of vaccination coverage refers only to the participants who are eligible. All pediatric patients were assessed for annual influenza and SARS-CoV-2 vaccination and for most of the basic vaccines including hepatitis A (hepA), hepatitis B (hepB), *Streptococcus pneumoniae*, tetanus–diphtheria–pertussis (DTaP or Tdap), measles–mumps–rubella (MMR), varicella–zoster (VZV or VAR), meningococcus serotype C (MCC or Men C), serotype B (Men B), ACWY (MCV4), and HPV. Adult patients with CKD were assessed for important vaccines for hemodialysis such as hepatitis B and A, *Streptococcus pneumoniae*, tetanus–diphtheria, HPV, and herpes zoster and annual influenza and SARS-CoV-2 vaccination. Adult relatives of children with CKD were asked about booster tetanus–diphtheria, annual influenza, and SARS-CoV-2 and *Streptococcus pneumoniae* vaccination for those who were eligible due to age or comorbidity. We chose not to estimate RSV vaccination in adult close contacts due to their younger ages.

### 2.5. Statistical Methods

The vaccination rates and 95% confidence intervals were calculated with the method of Clopper Pearson and were expressed as percentages of vaccinated over total participants for each group. Continuous variables (age) are described with percentages, mean and standard deviation. For comparisons between groups the chi-square and Fisher exact tests with Bonferroni corrections for multiple testing were used through the SPSS Statistics 27 statistical program, through which sample size and power of comparisons were also evaluated.

## 3. Results

### 3.1. Vaccination of Children with Chronic Kidney Disease

**Figure 2:**
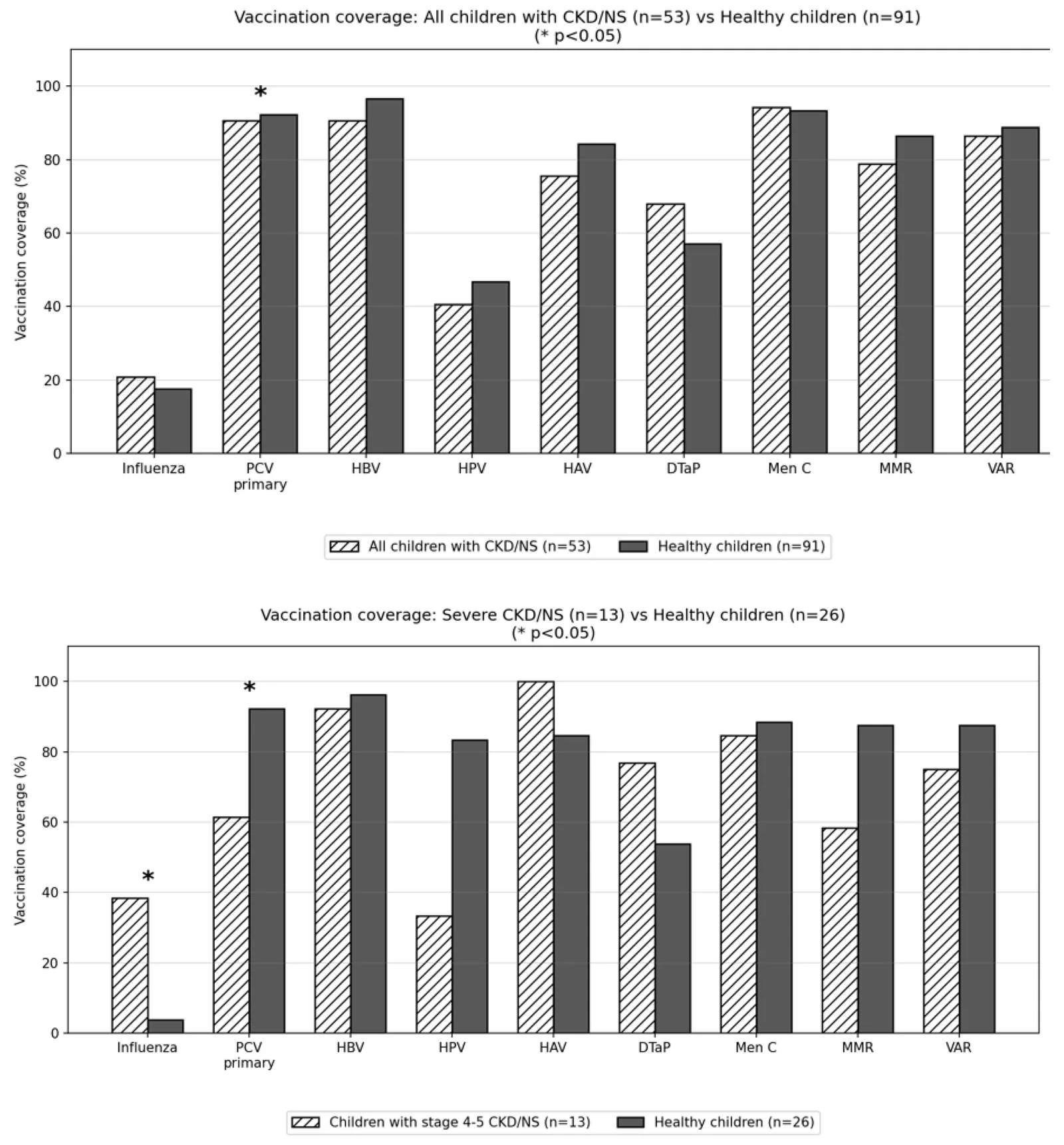
Vaccination coverage of children with CKD, healthy controls and comparison of groups.

Out of the 264 patient visits in the Pediatric Nephrology Outpatient Clinic, 53 patients were eligible for selection in the CKD children group according to the preset criteria. The mean age of the group was 9.48 years (SD 4.53), and 52% of the patients were male. A significant percent (25%) reported a history of immunosuppressive therapy, which could affect their present vaccination status. The most common underlying pathology was unilateral kidney agenesis or acquired solitary kidney (n = 15, 28.3%), followed by congenital anomalies of the kidney and urinary tract (CAKUT) (n = 14, 26.4%) (Table 1). There were 12 children with nephrotic syndrome (22.6%), while most patients had CKD stage 2 (45/52), four children had stage 3, one child had stage 4, and two patients had stage 5. Vaccination coverage was low for several important vaccines for children with CKD such as influenza (20.8%), COVID-19 booster (0%), additional pneumococcal vaccine (15.4%), MMR (78.8%) and VZV (85.5%). Children with CKD were adequately vaccinated for most vaccines of the basic immunization program except for DTaP/TdaP (67.9%), HPV (40.6%) and Men B (30.2%, 95% CI 18.3–44.3%). (Table 2 and Figure 2).

### 3.2. Comparison of CKD Children Group with Healthy Control Group

In total, 46/53 CKD patients (0–14 y) of the Pediatric Renal Outpatient Clinic were compared with 92 age- and sex-matched healthy controls, and this comparison was sufficient to detect a difference of 25% between groups with 80% power (a = 0.05). There were no statistically significant differences in vaccination coverage besides a marginally statistically significant difference in pneumococcal vaccination (80% in the patient group vs. 92.3% in the control group, *p* = 0.048). Both groups had extremely low vaccination coverage for influenza (17.6% in the control group, 26.7% in the CKD group) and relatively low vaccination rates for MMR, with 85.7% in the control group and 77.3% in the CKD group, and VZV, with 88.9% in the control group and 84.1% in the CKD group.

Further comparison of the 13 children with end-stage CKD or nephrotic syndrome, although underpowered (46%, a = 0.05), showed a better vaccination rate for influenza, with a statistically significant difference, than that of their 26 controls (38.5% vs. 3.8%, *p* = 0.008), but this group of children with CKD had the lowest percentages among other pediatric groups (CKD stages 2–5 or nephrotic syndrome, healthy controls and children from the environment of patients) for important vaccines such as HPV (33.3%), basic pneumococcal (61.5%, statistically significant difference from healthy controls—92.3%, *p* = 0.03), VZV (75%) and MMR vaccination (58.5%) (Table 1).

### 3.3. Vaccination of Adult Patients in Dialysis Unit

This patient group included all but one (63 out of 64) adult patients in the dialysis unit. The mean age was 66.4 years (SD 17), and 56.3% were male. A significant percentage (19%) were on immunosuppressive therapy, and 25.8% had diabetic nephropathy. The most common documented comorbidity was cardiovascular disease (41.3%), followed by malignancy (17.5%) and then rheumatic disease (7.9%), while the underlying kidney pathologies amongst the patients were solitary kidney (33%), glomerulonephritis (19%), polycystic kidney disease (3.2%) and Alport syndrome (1.32%). HD patients were inadequately vaccinated for every vaccine recorded in this study. More specifically, vaccination was low for influenza (61.9%), RSV (50%) and the COVID-19 booster (3.2%), whereas it was satisfactory (79.4%) for the initial doses. Despite the recommendations, vaccination rates were particularly low for *Streptococcus Pneumoniae* (25.4%) and HBV (41.3%). Nearly half (44.8%) of the participants had protective titres of Anti-HBs (>10 miU/L). (Table 3).

### 3.4. Vaccination of Close Relatives of CKD Patients

Vaccination of close relatives of patients with CKD focused on children close contacts of adult participants on hemodialysis (n = 30) and both adult (parents/grandparents, n = 108) and children (siblings, n = 35) close contacts of children participants in the study. All children close contacts were reported to be free of chronic health conditions and had no further contra-indications for vaccination. Among adult close contacts, 14 (12.9%) reported chronic health issues which could justify additional vaccination against *Streptococcus pneumoniae*. The vaccines that were studied in this group were not contra-indicated for immunocompromised patients. Influenza vaccination was lower than optimal in all relative groups. Vaccination coverage for the basic pediatric vaccination program was adequate for almost every vaccine, but adult relatives of children with CKD were inadequately vaccinated. The epidemiological features and the vaccination coverage of the groups are summarized in Table 4.

### 3.5. Tendencies of Vaccination Coverage

**Figure 3:**
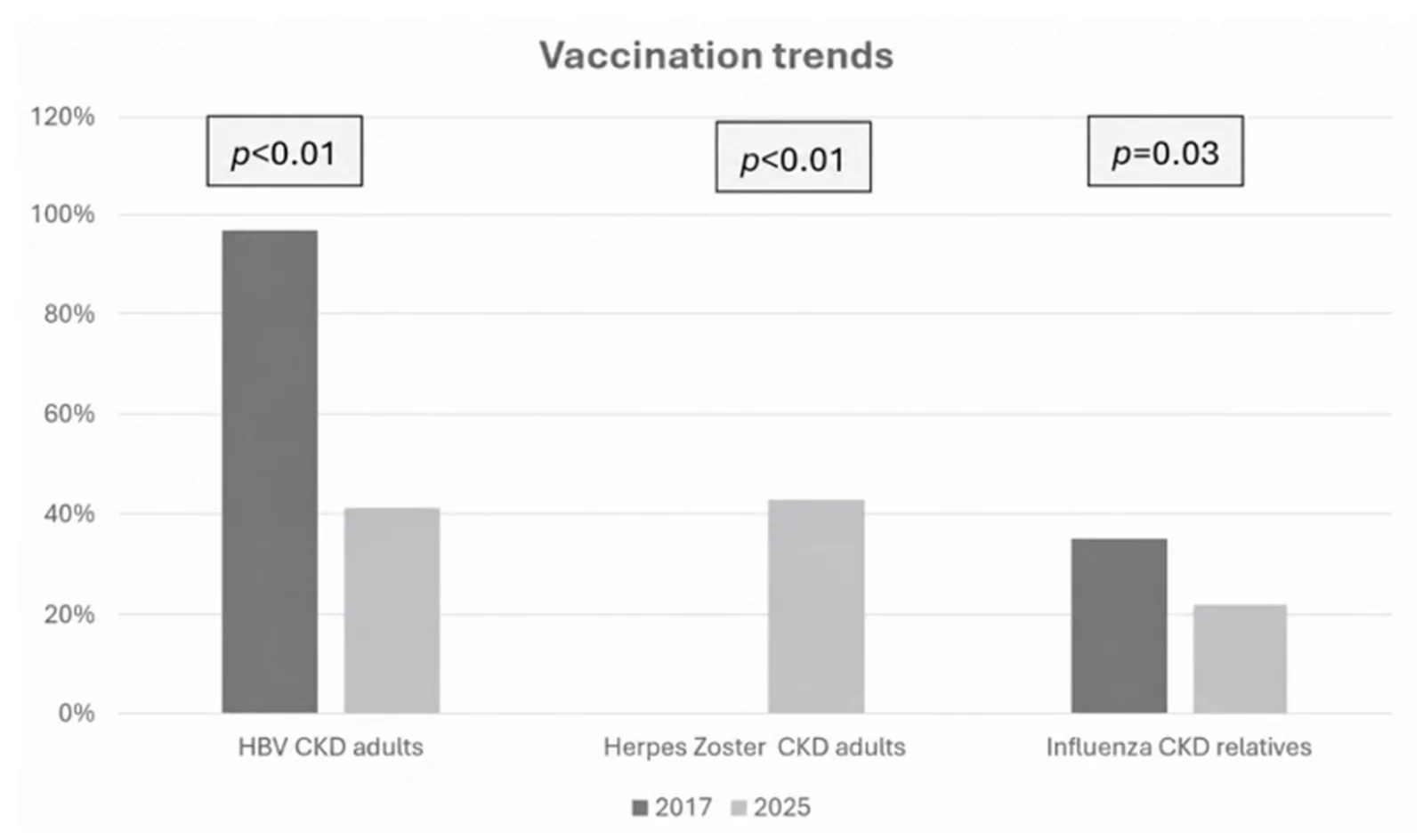
Major vaccination trends between 2017 and 2025 groups.

Τo investigate the potential effect of the COVID-19 pandemic on vaccination rates of patients with CKD, we compared the patient and relative groups of 2025 with groups from a similar study about vaccination coverage and the cocooning strategy for CKD patients, which was carried out in 2017 and included patients from the same departments of our hospital using a similar methodology of selection and data documentation [32]. The comparison of vaccination of children relatives of adult CKD patients and children with CKD showed no significant differences in vaccination between 2017 and 2025. Therefore, overall pediatric vaccination remained stable within the last eight years. Adult relatives of pediatric CKD patients were better vaccinated for influenza in 2025 (27.8% vs. 3% in 2017, *p* = 0.03). Adult CKD patients’ HBV vaccination dropped significantly (from 97% to 41.3% in 2025, *p* < 0.01), and a significant rise in vaccination for herpes zoster vaccine (from 0 to 42.9% in 2025, *p* < 0.01) was noted (Figure 3). Other comparisons of vaccination did not showcase significant differences in the adult population of the study.

## 4. Discussion

Our aim was to describe the vaccination coverage of patients with CKD, and the results of this study pinpoint towards inadequate immunization for almost every recorded vaccine amongst adult patients and for important vaccines for CKD, such as influenza, COVID-19 and additional pneumococcal vaccine in children. Vaccination rates of children with CKD did not differ significantly from those of healthy children in our area as was documented in our case–control study, but significantly superior influenza vaccination rates were found in children with severe CKD or nephrotic syndrome. Vaccination trends over time showed no significant differences in pediatric vaccinations. On the contrary, adult CKD patients’ vaccination rates dropped significantly post-pandemic for HBV and improved for herpes zoster. Our study is the first to assess cocooning strategy implementation in patients with CKD, and our findings suggest suboptimal immunization levels of patients’ adult close contacts for important vaccines for CKD patients such as influenza and COVID-19. However, children being sufficiently vaccinated for most vaccines in the basic vaccination program were more protective of their susceptible relatives.

Children with advanced CKD have clear recommendations for annual influenza and COVID-19 vaccinations, and supplementary pneumococcal vaccination is recommended for children with nephrotic syndrome [36]. Our study includes patients with earlier-stage CKD, a subgroup that is often under-represented in epidemiological vaccination coverage studies [3]. Despite the sufficient rates of basic pediatric vaccination, similar to those of healthy controls for almost every vaccine, our patients were not completely vaccinated according to current guidelines, as was the case in previous studies. Complete vaccination has been reported to be only 8.4% in a European multicenter study of children candidates for kidney transplant despite the severity of their disease [37]. Interestingly, our pediatric patients with advanced CKD and nephrotic syndrome were better vaccinated for influenza than the healthy controls.

Influenza vaccination coverage was lower compared to other reports in the literature, partly because of differences in methodology and inclusion of more end-stage patients (24–66%) with greater urgency for vaccination in previous studies [38,39,40]. The reported reasons for inadequate vaccination in our study included underestimation of the need to vaccinate or the severity of the child’s disease, doubts about the effectiveness of the vaccine, or unawareness of recommendation. Previous studies have showcased the importance of recommendation from the pediatrician or pediatric nephrologist, as well as parents’ education and perception of the severity of their child’s condition [38,41]. Insufficient COVID-19 vaccination in our study (0%) could be attributed to the gradual abandonment of the strict measures imposed during the pandemic and to the observed favorable prognosis after COVID-19 infection compared to adult patients. Furthermore, available data challenge the need for COVID-19 vaccination in pediatric CKD patients despite the higher hospitalization rates of these children [18,42].

Additionally, pediatric patients with CKD exhibited a tendency for lower vaccination rates for live vaccines such as MMR and VZV, especially children with nephrotic syndrome, which could be attributed to their immunosuppressive therapy. Tran et al., in their study of children with nephrotic syndrome, documented a low level of awareness of guidelines for live vaccines amongst caretakers and healthcare providers [43]. Kamei et al. who vaccinated selected pediatric patients with nephrotic syndrome based on immunological criteria deduced that MMR and VZV immunization could be safe and effective in children with mild or intermediate immunosuppression [44,45]. Current guidelines encourage an individualized approach based on the intensity of immunosuppressive regimen and the physician’s perception of the patient’s status [46].

The comparison between children with CKD and the healthy controls in our study demonstrated inferior immunization for pneumococcus among children with CKD and superior vaccination coverage for influenza among children with severe CKD. Until recently, national vaccination coverage studies were scarce as the previous national study ordered by the Greek Center of Disease Control was conducted in 2012 [47] and mostly relied on studies focused on certain age groups [22,48,49]. This could be attributed to the lack of electronic records on childhood vaccination and the vaccination of children in private practices. In 2024, a survey on vaccination coverage indicators based on the electronic prescription system records was published by the Greek Center of Disease Control [50]. The difference in methodology inhibits direct comparison with our results; however, a tendency of better vaccination coverage of our patient and control groups should be noted. Our patient–control cohort study, despite its limitations, could be indicative of the adequacy of vaccination coverage in CKD children in our area.

CKD patients undergoing hemodialysis are known to have a specific kind of immunosuppression with gradually reduced ability to respond to immunization [11]. Timely vaccination with a proper dose regimen for HBV and pneumococcal vaccination are of the utmost importance. Our study showcased a far from optimal vaccination status amongst hemodialysis patients. Vaccination coverage was subpar for influenza, COVID-19 and RSV, all of which are important viral air-borne pathogens which are associated with higher morbidity, hospitalization and mortality in CKD patients [17]. Although only 61.9% of the patients were vaccinated for influenza in the past two years, this rate is amongst the highest documented in similar populations in the literature [51,52,53,54,55]. COVID-19 vaccination was particularly low (3.2%), despite the high vaccination rates at the height of the pandemic (79.4%). Participants expressed a high level of hesitation and considered the initial doses of the vaccine to be responsible for the deterioration of their chronic condition. On the contrary, in a 2022 multicenter study, 78.2% of hemodialysis patients were positively predisposed towards the COVID-19 booster [56]. RSV vaccination rate was moderate (50%), despite the committed effort of the personnel of the hemodialysis unit, due to the recent introduction of the vaccine.

Hemodialysis patients were inadequately vaccinated for other important pathogens such as HBV and *Streptococcus pneumoniae*. The introduction of a 20-valent conjugated pneumococcal vaccine (PCV20) simplifies the vaccination schedule and is promising in terms of prevention of hospitalization; thus it is more cost-effective compared to older regimens [35]. Vaccination coverage surveys are lacking after the introduction of PCV20, but nonetheless vaccination in our study is lower than that in older similar studies (35.9–53%) [51,54]. Similarly, HBV vaccination was low (41.3%). Patients that undergo dialysis are susceptible to severe viral-mediated morbidity, either by reactivation of VZV (herpes zoster) or by the enforcement of oncogenic properties of HPV. These side effects are proportional to the level of immunosuppression and are accentuated in kidney transplant patients [57,58]. Vaccination in our study was 0% for HPV and 42% for herpes zoster. Finally, our patients had a significantly lower vaccination coverage for tetanus-diphtheria compared to other similar surveys (2.5–24%) [51,59,60].

The vaccination rates in our groups in the past eight years were surprisingly stable for the majority of the vaccines in pediatric CKD population despite the historical, societal and epidemiological differences between the two time periods. However, HBV vaccination declined in adult CKD patients in comparison to 2017. Hepatitis B carriage in hemodialysis units has been significantly reduced in advanced countries after the implementation of preventive measures such as immunization of the general population, sterilization techniques, timely diagnosis and isolation of infected patients during dialysis sessions [61]. The reduced perception of risk due to the decline in carriage has probably led to a decrease in the systematic effort to vaccinate patients with CKD. The introduction of the novel inactivated shingles vaccine enabled the immunization of a greater number of patients, which is depicted in the rise in vaccination by 42% in the hemodialysis unit patients of the present study.

Despite numerous studies focusing on the cocooning strategy for the protection of the neonatal population, there is a lack of data for patients with CKD or other chronic conditions, with the exception of a 2015 Polish study which recorded vaccination of children close contacts of patients with inflammatory bowel disease [45]. Our study documents the vaccination of both adults and children close contacts of CKD patients and compares their vaccination status in two different time periods in the same departments [32]. Adult close relatives of children with CKD were inadequately vaccinated for every vaccine that is indicated for adults of the general population, including influenza vaccination that was found to remain low (27.8%) but experienced a significant rise between 2017 and 2025. In the pediatric groups, immunization was adequate for most of the vaccines of the basic vaccination program. Influenza vaccination was the lowest amongst close contacts of patients in the dialysis unit as nephrologists are not authorized to prescribe vaccines to children. Men B vaccine was recently introduced in the mandatory vaccination schedule; thus older children were less likely to be vaccinated. Similarly, HPV vaccination was low due to the recent inclusion of boys in the mandatory vaccination. There is no specific provision for free vaccination of cohabitants of patients with CKD in our country, which is a potential barrier to the implementation of cocooning strategy in this population. The 2015 Polish study showed lower vaccination levels compared to our population [62].

Among the limitations of this study is its single-center basis, which limited the number of participants, as well as the representation of subgroups of patients with CKD. The representation of children with CKD and nephrotic syndrome could accurately indicate the vaccination coverage of children with CKD in Crete, but the results cannot be generalized to a national level. Similarly, the results of adult CKD patients cannot indicate the national vaccination coverage. Given the lack of data on vaccination rates of children with chronic kidney disease and the unique characteristics of this study, such as the report of the vaccination coverage of close contacts of patients with CKD, a vulnerable group that could benefit from the cocoon strategy, and the comparison of vaccination trends pre- and post-pandemic in the CKD patient population, the limitation of small numbers is somehow outweighed, and the study provides further insights into the field of vaccination of vulnerable populations. The data were retrieved from interviews with the patients, which could involve recalling bias as a possible methodological error. We attempted to mitigate this possibility by cross-checking our data with department archives and electronic records of the patients. Our study is the first to our knowledge to compare patients’ vaccination to healthy controls in the pediatric CKD population. However, we did not include a control group for the adult patients, and in the case of children with severe CKD, the limited number of patients weakened the comparisons. We experienced difficulties in accessing HD patients’ children relatives as the vast majority of the patients were not the primary caretakers and therefore were not always authorized to provide information on the vaccination status of the children. Finally, our study did not include confounding factors of vaccination such as education, socioeconomic status, duration of CKD, transplant candidacy or physician recommendation.

### Further Research Directions

Patients with chronic kidney disease experience increased infection-related morbidity and mortality, and vaccination of patients and their close contacts could protect them from exposure to disease and infectious complications. However, review of the literature reveals decreased vaccination coverage, underestimation of its importance by both patients and their physicians, as well as institutional and organization barriers [12]. Our experience through this study reinforces this conclusion.

Documentation of vaccination in electronic registries has only recently been supported for pediatric patients in Greece (2022), and its use has only recently become mandatory. This hinders the frequent and accurate estimation of vaccination percentage; thus this deprives us of feedback of the success of our vaccination strategies. This is emphasized in our population that includes patients with CKD, an often underdiagnosed and under-documented condition. Actions on an institutional level (clinics, hemodialysis units) and on a state level with the extension of vaccine registries for adults and susceptible populations could significantly improve the data on vaccination. Future studies could include data from multiple centers, with a greater number of patients and include information on vaccine hesitancy, immunogenicity and barriers towards vaccination.

## 5. Conclusions

Our study confirms that vaccination coverage among CKD patients remains suboptimal despite strong recommendations and clear evidence of benefit. Immunization towards important pathogens such as COVID-19, influenza, pneumococcus and, in the case of adult patients, HBV was found to be inadequate, and the cocooning strategy, despite its benefits, has not been properly implemented in our center. The reduction in HBV vaccination in end-stage CKD patients in the past 8 years along with the persistently low levels of influenza vaccination in all study groups command our attention. The proper documentation of vaccination rates, possibly in the form of national electronic registries or in terms of department organization, could provide valuable insight and aid the clinicians in their quest to properly vaccinate their patients. The implementation of the cocooning strategy is promising not only in terms of protecting susceptible individuals, but also in weaponizing love and altruism to increase overall vaccination rates. This enforces a positive correlation and enhances the public’s positive perceptions on vaccination. Ultimately, vaccination is a valuable and tested preventive strategy that should not be overlooked in susceptible populations.

## Figures and Tables

**Table 1:** Definitions for full vaccination coverage (according to Greek National Immunization Schedule of 2025).

Vaccine	Number of Doses	Ages and Doses for Healthy Children	Extra Doses for Children with CKD	Recommended Doses for Adults Undergoing Hemodialysis
HepB	3–4	2, 4, 6 mo	Stage 4–5 annual Anti-HBs monitoring, repeat regimen if Anti-HBs < 10IU (according to local protocol)	Annual Anti-HBs monitoring, repeat regimen if Anti-HBs < 10IU (according to local protocol)
DTaP (<7 y)	5	2, 4, 6, 15–18 mo, and 4–6 yrs	-	-
Tdap/Tdap-IPV	1	11–12 yrs	-	1 booster/10 y
PCV	3	2, 4, 12 mo (2+1 regimen)	Additional dose of PCV20 if prior vaccination was administered with PCV13 or PCV20 2+1 regimen	1 dose of PCV20 (timing depends on whether they had received previous pneumococcal vaccination)
MCC	1	10 mo	-	-
MCV4	1	11–12 y	-	-
Men B	3	2, 4 mo + booster at 12–15 mo	-	-
MMR	2	12–15 mo and 24–35 mo	-	-
VZV	2	12–15 mo and 24–35 mo	-	-
HepA	2	2 doses (2–6 y)	-	2 doses (if unvaccinated)
HPV	2 or 3	9–11 y (3 doses in high-risk)	3 doses in high-risk groups	18–45 y (3 doses if unvaccinated)
Influenza	1/year (2 for first-time vaccinees <9 y)	Annually (6 mo–5 y)	Annual	Annual
COVID-19	1 (≥5 y) or 3 (6 mo–4 y)	High-risk groups only	1 dose of the COVID-19 vaccine (Booster) if >5 y, or 3 doses if 6 mo–4 y	1 dose
RSV	-	-	-	1 dose (All ≥ 75 y, Patients with CKD stage 4–5 over 60 y)
**Differences between 2017 and 2025 Greek Vaccination Program:** PCV13 4 (3+1) doses vs. PCV20 (2+1) Influenza vaccination only in susceptible groups vs. vaccination of every child between the ages of 6 mo and 5 y and the susceptible groups Men B vaccination only in high-risk groups vs. Men B in every infant Live vaccine for herpes zoster vaccination only for adults over 60 y vs. conjugated in adults over 60 y and susceptible individuals over 18 y PCV13 and polysaccharide (PPSV) regimen for high-risk populations vs. an additional dose of PCV20 HPV vaccination only for girls vs. HPV vaccination for boys and girls since 2022 RSV and COVID-19 vaccines were not invented in 2017				

Abbreviations: HepB: hepatitis B; DTaP: diphtheria, tetanus, pertussis; HepA: Hepatitis A; HPV: human papillomavirus: MCC: meningococcal conjugated vaccine for serotype C, MCV4: meningococcal conjugated vaccine for serotypes ACWY; MMR: measles, mumps, rubella; Tdap: tetanus, diphtheria, pertussis; PCV: pneumococcus conjugate vaccine; PPSV: polysaccharide pneumococcal vaccine; RSV: respiratory syncytial virus.

**Table 2:** Vaccination coverage of children with CKD, healthy controls and comparison of groups.

Vaccine	Children with CKD, or NS (n = 53), (%)	95% CI (%)	Healthy Controls (n = 91), (%)	95% CI (%)	P^1^ *
Influenza	20.8	10.8–34.1	17.6	10.4–27	0.261
COVID-19 upd	0	0–8	-	-	-
PCV basic	90.6	79.3–96.9	92.3	84.8–96.9	**0.048**
PCV addit.	15.4 (2/13)	1.9–45	-	-	-
HBV	90.6	79.3–96.3	96.7	90.7–99.3	0.219
HPV	40.6 (n_tot_ = 32)	23.7–59.7	46.7 (21/45)	32.9–63.1	0.312
HAV	75.5	61.7–86.2	84.3	74.3–90.5	0.267
DTaP	67.9	53.7–80.1	57.1	46.3–67.5	0.265
Men C	94.3	84.3–98.3	93.3	89–98.8	1
MMR	78.8 (n_tot_ = 52)	65.3–88.9	86.5	76.8–90.2	0.216
VAR	86.5 (n_tot_ = 52)	74.2–94.4	88.8	80.5–94.5	0.582
**Vaccine**	**Children with CKD Stages 4–5 or NS (n = 13)**	**95% CI** **(%)**	**Healthy Controls** **(n = 26)**	**95% CI** **(%)**	**P^2^**
Influenza	38.5	15.2–72.3	3.8	0.01–19.1	**0.008**
COVID-19 upd	0		-	-	-
PCV basic	61.5	31.6–86.1	92.3	74.9–99.1	**0.03**
PCV addit.	15.4	1.9–45	-	-	-
HBV	92.3	64–99.8	96.2	80.4–99.9	1
HPV	33.3% (2/6)	23.7–59.7	83.3 (10/12)	41.9–91.6	0.074
HAV	100 (12/12)	61.7–86.2	84.6	65.1–95.6	0.296
DTaP	76.9	46.2–95	53.8	33.4–73.4	0.163
Men C	84.6	54.6–98.1	88.5	74–99	1
MMR	58.3 (7/12)	27.7–84.8	87.5 (21/24)	65.1–95.6	0.086
VAR	75 (9/12)	42.8–94.5	87.5 (21/24)	68.8–97.5	0.640

* Comparison with p1 refers to the comparison of 45 healthy children with 91 age- and sex-matched controls. Abbreviations: COVID-19 upd: vaccination with the booster doses of COVID-19 vaccine, PCV basic: pneumococcal vaccination indicated for general population, PCV addit.: additional recommended dose for children with advanced kidney disease or nephrotic syndrome, HBV: hepatitis B virus, HAV: hepatitis A virus, HPV: human papillomavirus, DTaP: diphtheria–tetanus–pertussis, Men C: Meningitis serotype C, Men B: Meningitis serotype B, MMR: measles–mumps–rubella, NS: nephrotic syndrome, VAR: varicella–zoster virus.

**Table 3:** Vaccination of adult hemodialysis patients in 2025 and comparison to 2017.

Vaccine	2025	95% CI	2017	95% CI	*p*
Influenza	61.9% (n = 39)	49–74	51.4% (n = 35)	39–62	0.290
COVID-19 first dose	79.4% (n = 50)	67–88	-	-	-
COVID-19 booster	3.2% (n = 2)	0–11	-	-	-
RSV	50% (n = 22)	34–65	-	-	-
PCV	25.4% (n = 16)	15–37	19.1% (n = 13)	11–30	0.408
HBV	41.3% (n = 26)	29–54.4	97% (n = 66)	89–99	**<0.01**
Anti-Hbs >10	44.8% (n = 25)	31–58	-	-	-
HPV (n = 6)	0%	0–45	-	-	-
HepA	1.6 (n = 1)	0–85	-	-	-
Td	3.2(n = 2)	0–11	4.5% (n = 3)	0.01–12	1
Herpes Zoster	42.9 (n = 27)	44–69.5	0% (n = 0)	0–0.89	**<0.01**

Abbreviations: PCV: pneumococcal vaccine, HBV: hepatitis B virus, HAV: hepatitis A virus, HPV: human papillomavirus, Td: diphtheria–tetanus, Men C: meningococcus serotype C, Men B: meningococcus serotype B, MMR: measles–mumps–rubella, Anti-HBS: Anti-HBS antibodies.

**Table 4:** Vaccination of close contacts of CKD patients.

	Relatives of Patients of the Dialysis Unit (0–18 Years), n = 30		Relatives of the Pediatric Nephrology Outpatient Clinic (0–18 Years), n = 35		Adult Relatives of the Pediatric Nephology Outpatient Clinic, n = 108		
Vaccine	Vaccination Rate	95% CI	Vaccination Rate	95% CI	Vaccine	Vaccination Rate	95% CI
Epidemiologic features	Mean age: 9.58 y SD: 4.038 Male: 50%		Mean age: 12.27 y SD: 4.22 Male: 57%		Mean age: 46.8 y SD: 13.8 Female: 51.7%		
Influenza	13.30% (n = 4)	3.8–30.7	22.90% (n = 8)	10.4–40.1	Influenza	27.8% (n = 30)	19.6–37.2
PCV	100% (n = 30)	88.4–100	94.3% (n = 33)	80.8–99.3	COVID-19	1.9% (n = 2)	2–6.5
HBV	100% (n = 30)	88.4–100	97.1% (n = 34)	85.1–99.9	Tdap/Td	1.9% (n = 2)	2–6.5
HAV	96.7%(n = 29)	88.4–100	85.7% (n = 30)	29.4–67.5	PCV (n = 14)	57.1% (n = 8)	28.9–82.3
HPV	42.1% (n = 8)	20.3–66.5	48.3% (n = 16)	69.7–95.2			
DTaP/Td	80% (n = 24)	61.4–92	80% (n = 28)	63.1–91.6			
Men C	96.7% (n = 29)	82.8–99.9	97.1% (n = 34)	85.1–99.9			
Men B	50%(n = 15)	31.3–68.7	31.4% (n = 11)	8.7–49.1			
MCV4	90.9% (n = 29)	58–99.8	90.5% (n = 31)	69.6–98.8			
MMR	93.3% (n = 28)	77.9–99.2	94.30% (n = 33)	80.8–99.3			
VAR	90% (n = 27)	73.5–07.9	91.4% (n = 32)	76.9–98.2			

Abbreviations: PCV: pneumococcal vaccine; HBV: hepatitis B virus; HAV: hepatitis A virus; HPV: human papillomavirus; DTaP or Tdap/Td: diphtheria–tetanus–pertussis; Men C: meningitis serotype C; Men B: meningitis serotype B; MCV4: conjugate vaccine for meningococcus serotype A, C, W, Y; MMR: measles–mumps–rubella; VAR: varicella–zoster virus.

## Data Availability

The original contributions presented in this study are included in the article. Further inquiries can be directed to the corresponding author.

## Abbreviations

CAKUT	Congenital anomalies of the kidney and urinary tract
CKD	Chronic kidney disease
DTaP or Tdap	Diphtheria-tetanus–pertussis vaccine
HAV or HepA	Hepatitis A virus
HBV or HepB	Hepatitis B virus
HD	Hemodialysis
HPV	Human papillomavirus
Men B	Meningococcus serotype B
Men C or MCC	Meningococcus serotype C
MMR	Measles–mumps–rubella
VZV or VAR	Varicella–zoster virus
